# Shingles (Herpes Zoster) Mimicking Acute Abdomen

**DOI:** 10.7759/cureus.10762

**Published:** 2020-10-01

**Authors:** Sherif Monib, Emre Pakdemirli

**Affiliations:** 1 Breast Surgery, West Hertfordshire Hospitals NHS Trust, St. Albans and Watford General Hospitals, London, GBR; 2 Radiology, West Hertfordshire Hospitals NHS Trust, St. Albans City Hospital, London, GBR

**Keywords:** shingles, varicella zoster virus, shingles, herpes zoster, varicella zoster virus, acute abdomen, herpes zoster, abdominal rash, acute abdomen

## Abstract

Shingles is a very well-known viral infection caused by the varicella-zoster virus leading to painful skin rash. Although shingles can occur anywhere, it most often appears as a single stripe of blisters around the hypochondriac region. We are presenting a rare atypical presentation of shingles, as our patient presented with a picture of the acute abdomen a couple of days prior to the eruption of skin lesions.

## Introduction

Varicella-zoster virus (VZV) is a member of the α-herpesviruses subfamily and is a member of the Varicellovirus genus [1]. It can affect patients of any age, but primary VZV infection occurs during childhood leading to Varicella (chickenpox). Any reduction in the ability of an appropriate immune response could lead to reactivation of VZV from latency allowing the virus to travel anterogradely from the sensory ganglia to the skin nerve terminals and spreading to skin epithelial cells leading to the clinical signs of herpes zoster (HZ) [2].

It is usually a minor illness but can result in severe life-threatening complications in previously healthy patients. Herpes zoster rash usually involves a single dermatome and does not cross the midline. Thoracic, trigeminal, lumbar, and cervical dermatomes are the most frequent sites, but any area of the skin can be involved [3]. In rare cases, herpes zoster can present with atypical manifestations, like glioma, zoster sine herpete, and bilateral herpes zoster.

## Case presentation

We are presenting a case of a 53-year-old woman who was blue lighted from her workplace to the local accident and emergency department with severe, unbearable burning left upper quadrant pain radiating to the left lower thoracic region.

Her past medical history included indigestion and regurgitation under investigations, with no history of previous acute illness, similar conditions, or hospitalization. Her family history was irrelevant, the general examination was unremarkable, and her vital signs were within the normal range. Abdominal examination revealed a very tender, warm left hypochondrial region with hypersensitivity, with no organomegaly or visible skin lesions. Her ECG, as well as laboratory investigations including troponin level, were normal; her urine sample showed microscopic haematuria; a CT of the kidneys, ureters, and bladder (KUB) was carried out, which did not reveal any abnormality.

Due to the fact that she was hemodynamically stable and her biochemistry, as well as radiological investigations, were normal, she was discharged from the accident and emergency department with a provisional diagnosis of non-specific abdominal pain, with advice to see her general practitioner (GP) the following day for follow up as well as gastroenterology referral for investigation of indigestion and regurgitation.

**Figure 1:**
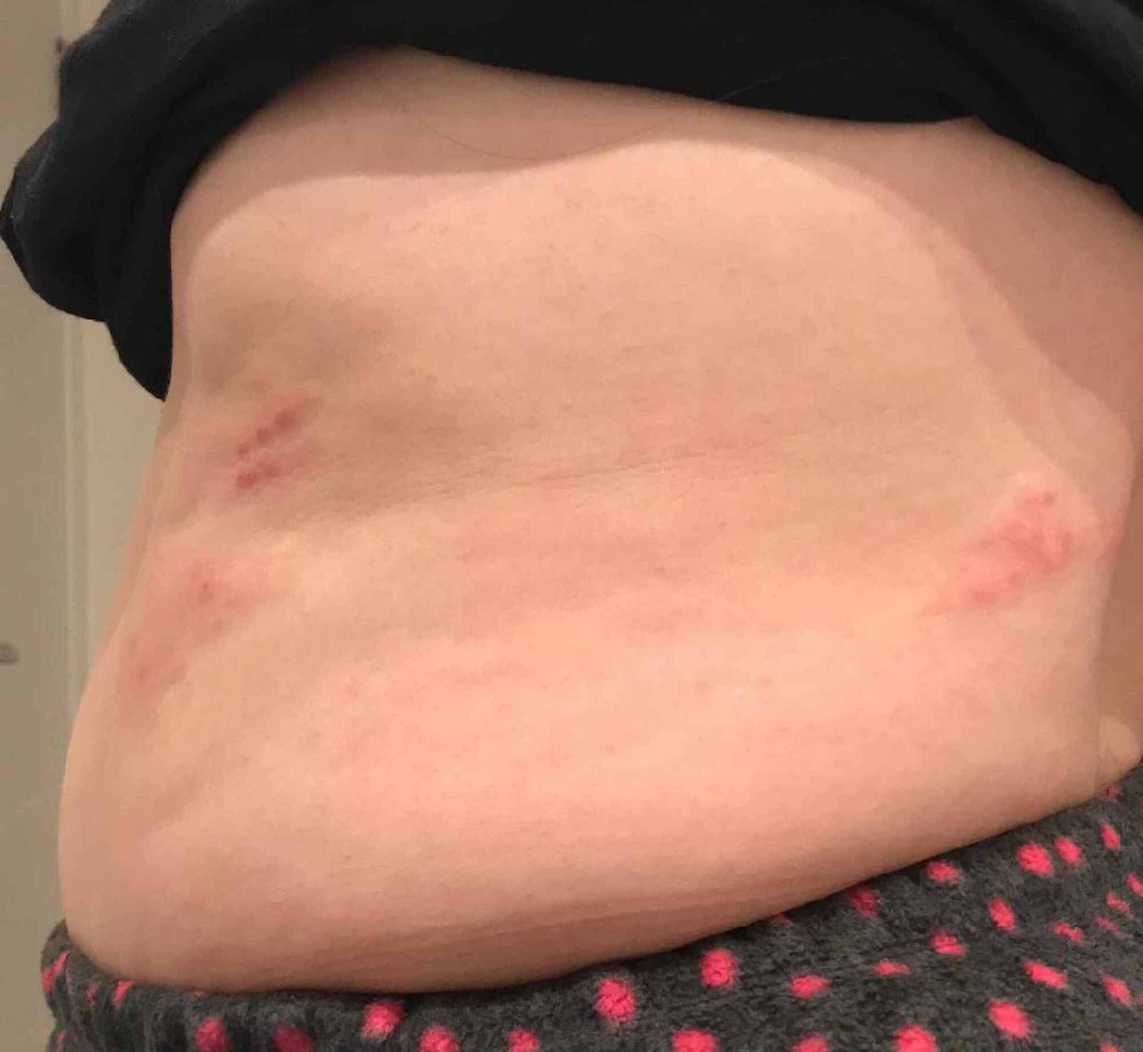
Clinical photograph of the patient's abdomen Involved patchy areas noted in the patient's abdomen and loin.

Twenty-four hours later, she developed left hypochondrial/loin dry skin rashes (Figure 1); her GP provisional diagnosis was shingles based on her clinical picture. Subsequently, she was started on oral Solpadol® (paracetamol 500 mg + codeine phosphate hemihydrate 30 mg) four times a day and oral acyclovir 800 mg five times a day. She eventually fully recovered a week after without any complications; clinical examination six weeks after revealed a complete resolution of symptoms.

## Discussion

Varicella-zoster virus (VZV) was initially isolated in 1954 from the vesicular fluid of both chickenpox and zoster lesions in cell culture by Thomas Weller. In 1970 a live attenuated varicella vaccine was established in Japan [1].

VZV causes two clinically distinct diseases - a primary infection result in varicella (chickenpox), which is an extremely contagious acute infection that occurs mainly in school-aged children and is characterized by a generalized vesicular rash. Like other α-herpesviruses, VZV establishes latency in neural tissue following primary infection. Reactivation of latent VZV from dorsal root ganglia leads to herpes zoster (shingles), which presents as localized cutaneous eruption associated by neuralgic pain that occurs most commonly in older patients. The typical clinical presentations of varicella and herpes zoster are easily recognized by most clinicians. In contrast, atypical clinical presentations and uncommon complications can be quite challenging not only to diagnose but also to treat [4].

The lifetime risk of herpes zoster is about 32%. Increasing age and cellular immunosuppression are the most important risk factors - 50% of persons living until age 85 years will develop it [4]. Herpes zoster infection is an interesting disease that involves many medical specialties including infectious diseases, dermatology, immunology, and neurology, while in most cases of HZ is a straight forward clinical diagnosis, an atypical presentation can be associated with a variety of severe and potentially lethal complications in both immunocompetent and immunocompromised persons. Therefore a low threshold of suspicion and early immunofluorescence assay for VZV antigen or polymerase chain reaction (PCR) assay, followed by adequate treatment involving different specialties as well as expertise in pain management and psychological support is required to reach a good outcome [5, 6].

Herpes zoster vaccine reduces the occurrence of the disease by about 50%. The efficacy of the vaccine was found to be highest at 64% in people aged 60-69, but unfortunately, its effectiveness declined with age - to 41% for people aged 70-79, and 18% for those 80 years of age and older [7]. 

While most patients' symptoms settle down within a week with only simple analgesia, others might need antiviral medications like acyclovir, valacyclovir, famciclovir, and brivudine [8].

## Conclusions

Atypical presentation of herpes zoster can lead to delay of diagnosis as well as the unfavorable outcomes. Herpes zoster infection should always be in mind when investigating patients presenting with acute onset of abdominal pain. We encourage reporting atypical cases of herpes zoster to create the opportunity to study different presentations and improve treatment modalities as well as outcomes.
